# Supplementary figures and images for: Overexpression screen of interferon-stimulated genes identifies RARRES3 as a restrictor of Toxoplasma gondii infection
Source: eLife. 2021 Dec 6;10:e73137. doi: 10.7554/eLife.73137 (PMC8789288; doi:10.7554/eLife.73137)

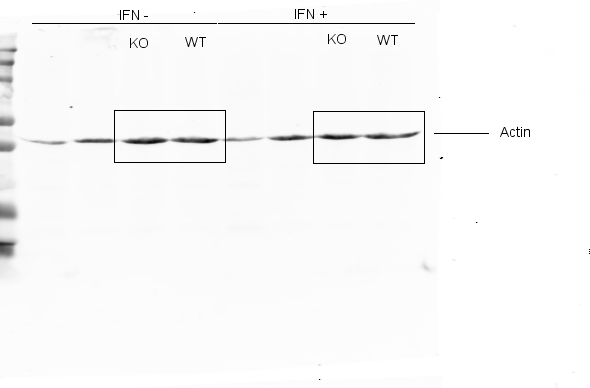

Supplement: Figure 1—source data 6. [file elife-73137-fig1-data6.zip › Figure 1 - Source Data 6.tif]

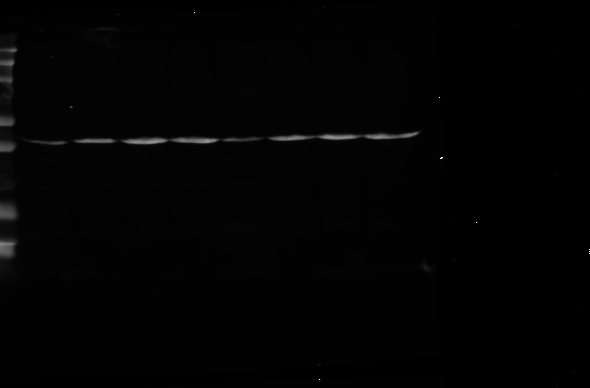

Supplement: Figure 1—source data 7. [file elife-73137-fig1-data7.zip › Figure 1 - Source Data 7.tif]

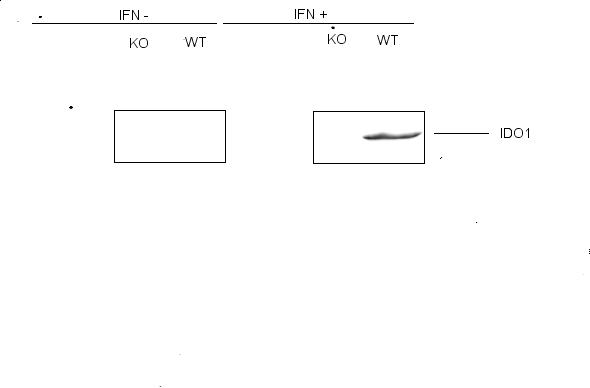

Supplement: Figure 1—source data 8. [file elife-73137-fig1-data8.zip › Figure 1 - Source Data 8.tif]

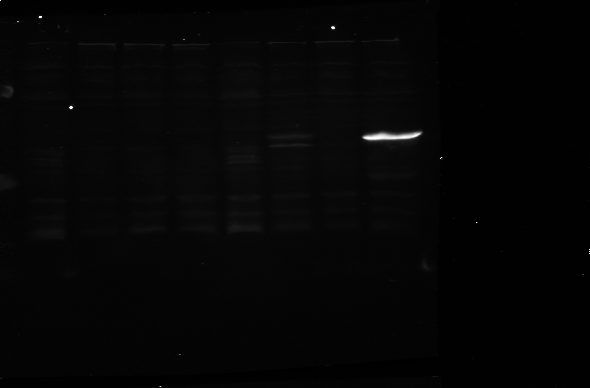

Supplement: Figure 1—source data 9. [file elife-73137-fig1-data9.zip › Figure 1 - Source Data 9.tif]

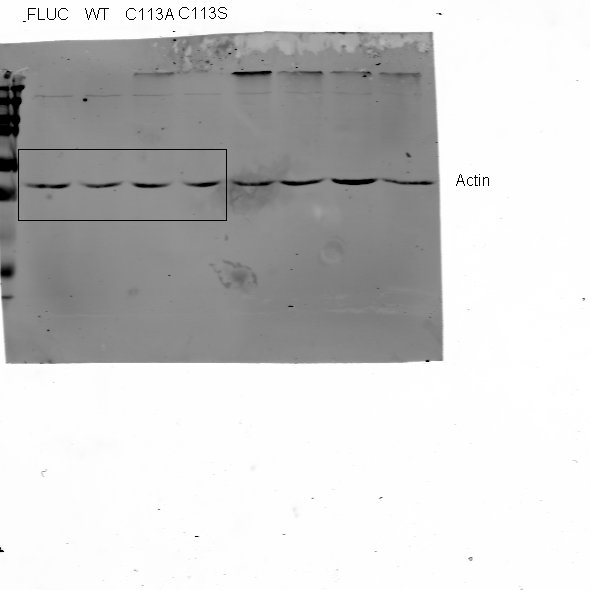

Supplement: Figure 5—source data 2. [file elife-73137-fig5-data2.zip › Figure 5 - Source Data 2.jpg]

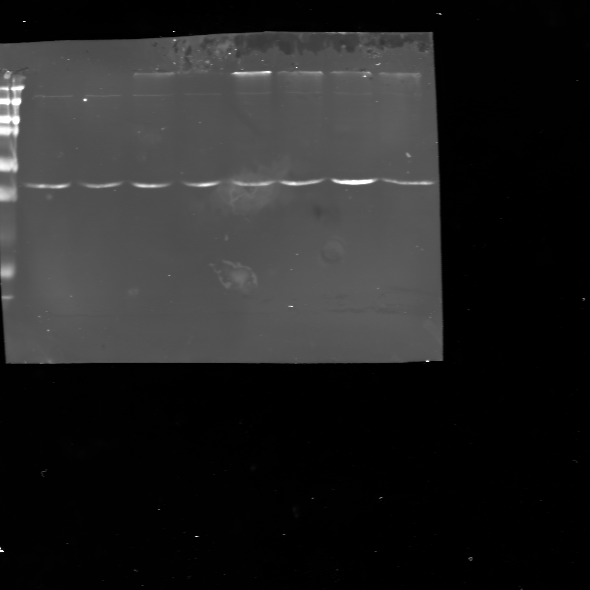

Supplement: Figure 5—source data 3. [file elife-73137-fig5-data3.zip › Figure 5 - Source Data 3.tif]

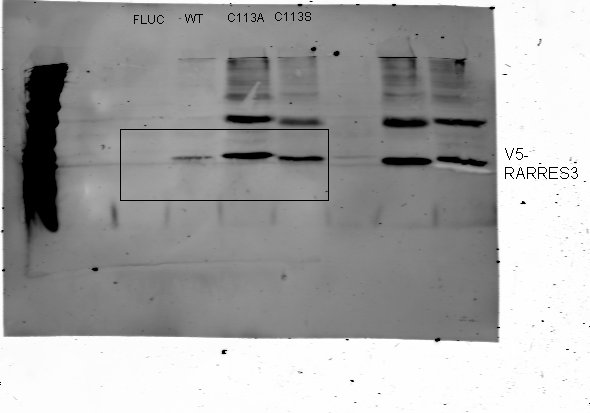

Supplement: Figure 5—source data 4. [file elife-73137-fig5-data4.zip › Figure 5 - Source Data 4.jpg]

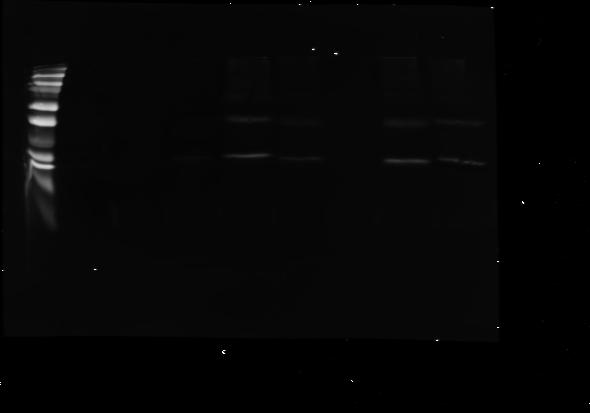

Supplement: Figure 5—source data 5. [file elife-73137-fig5-data5.zip › Figure 5 - Source Data 5.tif]
