# Supplementary material for: Overexpression screen of interferon-stimulated genes identifies RARRES3 as a restrictor of Toxoplasma gondii infection
Source: eLife. 2021 Dec 6;10:e73137. doi: 10.7554/eLife.73137 (PMC8789288; doi:10.7554/eLife.73137)
Supplement: Supplementary file 4. [file elife-73137-supp4.docx]

**Supplementary file 4.** **List of primer sets used.**

| Purpose | Forward Primer (5’->3’) | Reverse Primer (5’->3’) |
| --- | --- | --- |
| RARRES3 sgRNA #1 cloning | CACCGtgagtaccaaccacggcccg | AAACcgggccgtggttggtactcaC |
| RARRES3 sgRNA #2 cloning | CACCGcgtagccatctcctatatac | AAACgtatataggagatggctacgC |
| RARRES3 genomic amplification | AGTGAGAGTGCCTCTGATTGC | ACTCTCAGCCAGCAGGTCTC |
| STAT1 sgRNA cloning | CACCAAAAGAGGTCTCAATG | AAACCATTGAGACCTCTTTTC |
| STAT1 genomic amplification | CATGGCGCTAAATAGGCTTC | ATATGAGACAATGAGGAACGGA |
| Nontargeting sgRNA cloning | CACCGGTATTACTGATATTGGTGGG | AAACCCCACCAATATCAGTAATAC |
| IDO1 sgRNA cloning | CACCGATCCCAGAACTAGACGTGCA | AAACTGCACGTCTAGTTCTGGGATC |
| Cas9 resistant RARRES3 fragment 1 | CAGCTTGGACCAtgaatatcaacctcgccctgtggaggtgatcatcag | ctgatgatcacctccacagggcgaggttgatattcaTGGTCCAAGCTG |
| Cas9 resistant RARRES3 fragment 2 | CAGCTTGGACCAtgaatatcaacctcgccctgtggaggtgatcatcag | ggggaccactttgtacaagaaagctgggttTCAGGCTGTTGCTTTTTTTTGG |
| WT V5-RARRES3 | ggggacaagtttgtacaaaaaagcaggcttcaccATGGGCAAGCCCATCCCCAACCCCCTGCTGGGCCTGGACAGCACCGCTTCGCCACACCAA | ggggaccactttgtacaagaaagctgggttTCAGGCTGTTGCTTTTTTTTGG |
| V5-RARRES3 C113A fragment 1 | ggggacaagtttgtacaaaaaagcaggcttcaccATGGGCAAGCCCATCCCCAACCCCCTGCTGGGCCTGGACAGCACCGCTTCGCCACACCAA | CAAAGTGCTCAGCGTTCCTGCTC |
| V5-RARRES3 C113A fragment 2 | GAGCAGGAACGCTGAGCACTTTG | ggggaccactttgtacaagaaagctgggttTCAGGCTGTTGCTTTTTTTTGG |
| V5-RARRES3 C113S fragment 1 | ggggacaagtttgtacaaaaaagcaggcttcaccATGGGCAAGCCCATCCCCAACCCCCTGCTGGGCCTGGACAGCACCGCTTCGCCACACCAA | caaagtgctcagagttcctgctc |
| V5-RARRES3 C113S fragment 2 | gagcaggaactctgagcactttg | ggggaccactttgtacaagaaagctgggttTCAGGCTGTTGCTTTTTTTTGG |
